# Rituximab Therapy for Primary Sjögren’s Syndrome

**DOI:** 10.3389/fphar.2021.731122

**Published:** 2021-09-02

**Authors:** Yi Han Chen, Xin Yu Wang, Xin Jin, Zi Yang, Jianguang Xu

**Affiliations:** Key Laboratory of Oral Disease Research of Anhui Province, Stomatologic Hospital AndCollege, Anhui Medical University, Hefei, China

**Keywords:** rituximab, primary Sjögren’s syndrome, B-cell depletion, anti-CD20 therapy, B cell repopulation

## Abstract

Primary Sjögren’s syndrome (pSS) is a systemic autoimmune diseases of the connective tissues, characteristic of the presentation of keratoconjunctivitis sicca and xerostomia. A cardinal pathogenetic feature of SS is B-cell hyperactivity, which has invited efforts on optimal B-cell targeted therapy, whereas conventional corticosteroids and disease-modifying antirheumatic drugs (DMARDs) are restricted to symptomatic relief. As per the first EULAR recommendation for pSS patients published in 2020, regimens with monoclonal antibodies targeting B cells may be initiated in patients with severe, refractory systemic disease, notably rituximab (RTX), a mouse-derived monoclonal antibody that targets CD20 antigen and contributes to B-cell depletion. Nonetheless, the data available from clinical trials with RTX are often controversial. Despite the lack of promising results from two large RCTs, several positive clinical efficacies were demonstrated. This current review addressed the efficacy and safety of clinical trials available and elucidated the potential of RTX on the immune system, especially B and T cells. Furthermore, plausible explanations for the discrepancy in clinical data were also presented.

## Introduction

Primary Sjögren’s syndrome (pSS) is quite common, with a prevalence of 0.1–0.6% in adult population, wherein the ratio of females to males is at least 9:1, with age average of 50 years on diagnosis (Mariette and Criswell, 2018). Primary Sjögren’s syndrome (SS) is presented with lymphocytic infiltration in the salivary and lacrimal glands, leading to dry symptoms, i.e. keratoconjunctivitis sicca and xerostomia (Mavragani and Moutsopoulos, 2014). Vaginal dryness in women, nonproductive cough, or swelling of the salivary glands may develop. Persistence of swollen salivary glands may be the initial manifestation of pSS. Systemic symptoms include joint pain, chronic fatigue and discomfort (Seror et al., 2011) as well as systemic manifestations (Seror et al., 2010).

PSS patients may biologically exhibit B-cell activation, such as serum polyclonal hypergammaglobulinemia, elevated free light chain levels, and autoantibody positivity of rheumatoid factor (RF), anti-Sjögren’s syndrome-related antigen A (SSA, or Ro) antibody (prevalence of 60–80%), and anti-Sjögren’s syndrome-related antigen B (SSB, or La) antibody (prevalence of 30–40%) (Gottenberg et al., 2013). The B cells in the salivary glands, or rather, the target organ of pSS, may occasionally constitute ectopic germinal centers (GCs). Additionally, pSS enhances the susceptibility to B-cell lymphoma in individuals, particularly in pSS patients comorbid with lymphoma, RA, and SL (Song et al., 2018).

Current clinical regimens for pSS are mainly focused symptomatically on keratoconjunctivitis sicca and systemically on broad-spectrum immunosuppression. As per the updated EULAR recommendations (Ramos-Casals et al., 2012), SS patients should be treated in a specialized center or in close cooperation with a specialized center, with a multidisciplinary regimen (Ramos-Casals et al., 2020). However, there is a conflict between the urgency for specifically targeted therapy in clinical practice and conventional symptomatic alleviation with glucocorticoids and disease-modifying anti-rheumatic drugs (DMARDs). Recently, improved knowledge of the disease heterogeneity, availability of biologics and better elucidation of pathogenic pathways all contribute to international well-controlled trials of pSS.

RTX is a chimeric antibody with specific binding to the CD20 antigen with expression on the majority of B-cell progenitors, and facilitates them to activate, proliferate, and differentiate. In addition, RTX is deemed to reduce the number of circulating B cells via complement-dependent cytotoxicity (CDC) and antibody-dependent cytotoxicity (Beers et al., 2010). RTX could serve as a first-line therapy in patients with severe autoimmune rheumatic diseases (AIRD) (Galarza et al., 2008). Beyond the implication in B-cell depletion, RTX appears to regulate T-cell responses in autoimmune diseases (Ciccia et al., 2013; Ciccia et al., 2014). However, despite the possible mechanism of RTX, the researches of the RTX efficacy in pSS are still controversial. Following two small-sample studies with satisfactory results, two subsequent larger randomized controlled trials negated the potency of RTX in removal of B cells in pSS. Therefore, there is discrepancy as to the efficacy of RTX therapy for pSS, which however brought forth some clinical, biological and histological improvements. In addition, several clinical trials are currently enroute for the feasibility of rituximab-belimumab sequential therapy in SLE and SS, indicating the potential prospects of RTX combination therapy.

This review addressed the current literature available on RTX treatment in pSS patients, considering the effectiveness and safety of the clinical and biological environment. This review also discussed the underlying mechanism of RTX on B and T cells and the plausible explanation behind possible clinical phenomena.

### EFFICACY OF RITUXIMAB AND THE MECHANISMS OF ACTION

**FIGURE 1:**
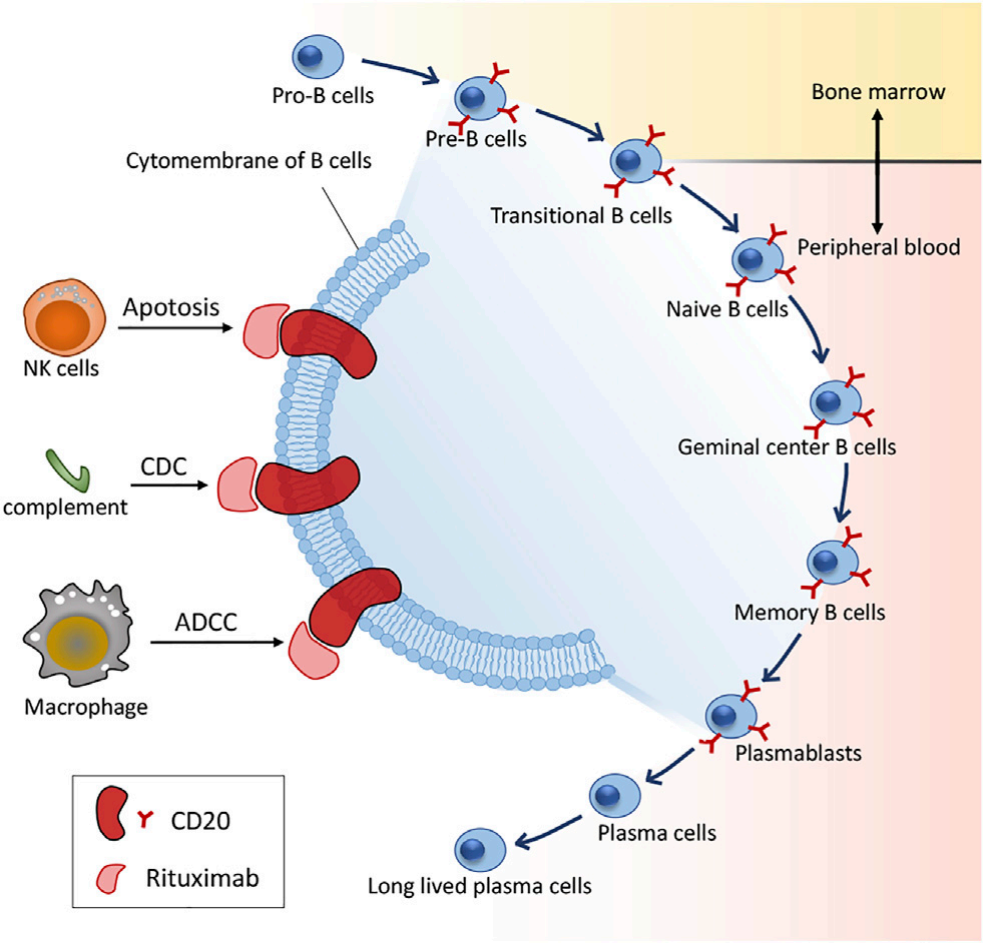
B cell differentiation and action mechanisms of RTX. CDC: complement-dependent cytotoxicity; ADCC: antibody-dependent cytotoxicity.

In 1997, RTX became the first approved mAb by the US FDA in regimens for relapsed/refractory non-Hodgkin’s lymphoma (NHL), and has thereon significantly benefited numerous patients with various autoimmune disorders, particularly B-cell malignancies, including pSS (Gürcan et al., 2009). CD20 (human cluster of differentiation 20) is an integral membrane protein expressed during the development of B lymphocytes (Einfeld et al., 1988) and is also the target of approved therapeutic monoclonal antibodies (mAbs) (Deans et al., 1998) with expression on the surface of normal and malignant B cells, wherein CD20 receptor is only absent in the pro-B lymphocyte and plasma cells (Perez-Callejo et al., 2015). RTX is a chimeric anti-CD20 type I recombinant monoclonal antibody with a mouse antigen binding domain connected to the constant domain of human immunoglobulin G1 (IgG1). There is a wealth of reports on the mechanisms of action of RTX as well as its effect *in vitro* in contrast to the obscurity *in vivo*. Honetheless, RTX may bind to its target CD20, thus rendering spatial reorganization of CD20 molecules in lipid rafts. Consequently, depletion mechanisms such as complement-dependent cytotoxicity (CDC), antibody-dependent cytotoxicity (ADCC) and phagocytosis of the reticuloendothelial system are activated, resulting in B-cell apoptosis via cross-linking CD20 molecules (Maloney et al., 2002) (Figure 1).

### B Lymphocytic Responses

PSS is characterized by B cell activation in pathogenesis. When RTX binds to CD20 molecules, the consequent cell lysis renders disappearance of B cells from the peripheral blood (PB). RTX therapy in pSS results in almost exhaustive depletion of B cells, with scanty CD19+ cells detected in PB (Meijer et al., 2010; St Clair et al., 2013). The effectiveness of RTX is mainly attributed to its effects on the production of plasmablasts via the depletion of their direct precursors, such as the activated B cells and germinal center. B cells are also proposed to produce antigen and secrete cytokine, the extra mechanisms contributing to the clinical efficacy of RTX (Barr et al., 2012). Nevertheless, inhibition of plasmablast production can account for the clinical effects of most B-cell-mediated morbidities, wherein the RTX efficacy is embodied by the gradual clearance of pathogenic antibodies and clinical remission. In addition, studies have confirmed that long-lived precursors can elicit the autoimmune response, which explains the failure of RTX to yield complete depletion of peripheral B cells and the presence of pathogenic autoantibodies detectable (Mei et al., 2015). We hypothesized that the inconsistency in clinical responses rendered by RTX might represent the heterogeneity of the generation of precursors. It would be of interest to reveal the microenviromental profiles in autoimmunity of both the long-lived and short-lived auto-reacting precursors and contribute to the development of novel regimens of specific targeting of these cells.

The suppression of pSS is implicated in long-term depletion of memory B cells, or biomarkers of disease activity in a number of CD20+ B cell depletion-sensitive autoimmune diseases. After B cells are depleted, the transitional (immature) and naïve (mature) B cells are rapidly regenerated in the bone marrow, which discounts the significant depletion via the slow regeneration of memory B cells derived from lymphoid tissue. Accordingly, short-term therapeutic cycles may render long-term protection, presumably via the stimulation of memory B cells by T cells to drive disease recurrence. Most of the B cells in PB come from bone marrow whereas the memory B cell response is gradually generated in the secondary lymphoid tissues.

In the clinical scenario, the effect of RTX therapy has been compared to “Road Block” (Silverman and Boyle, 2008). During the development of autoimmunity, autoreactive B cells have a central part to play in inflammation and pathogenesis via sequence transference to antigen-specific autoreactive B cells and BCR. Accordingly, Silverman and Boyle proposed that depletion of these autoreactive B cells should be able to annul the interaction between costimulatory signals and pro-inflammatory mediators (Silverman and Boyle, 2008). In this sense, blockade of the “road” of autoimmunity may contribute to the elimination of local inflammation and clinical recovery.

B-cell activating factor (BAFF) and proliferation-inducing ligand (april) play a central role in maturation, proliferation and survival of B cells. B cell-activating factor receptor (BAFF-r) is able to inhibit cell apoptosis via its affinity to BAFF, thereby occupying a pivotal part in B cell homeostasis (Schneider, 2005). Moreover, BAFF also serves as target factor for autoimmune diseases, with its level being subject to B cell population, i.e. a declined B cell counts may result in the elevation of free BAFF in the serum (Pollard et al., 2013). Likewise, a report on the mechanism of BAFF elevation after RTX medication also confirmed that the downregulation of BAFF receptors had a causative relation to the positive transcription regulation of BAFF and elevated serum BAFF protein and mRNA levels (Lavie et al., 2007). In addition, another study on RTX therapy for pSS described the attribution of the response to RTX to pretreatment BAFF levels and the B-cell activation that followed (Cornec et al., 2016a). Furthermore, patients with systemic lupus erythematosus (SLE), rheumatoid arthritis and pemphigus vulgaris usually present with elevated BAFF levels (Vallerskog et al., 2006; Nagel et al., 2009) and transgenic mice with the overexpression of BAFF developed a pSS-like pathology with age, manifested by infiltration of lymphatic tissue adjacent to the duct, destruction of glandular tissue, and diminished salivation (Groom et al., 2002). It seems that elevated BAFF levels may invalidate B-cell tolerance in pSS patients, and the combination of anti-BAFF and RTX therapy can result in long-term suppression of autoreactive B cells, whereas there is a paucity of evidence available.

Generally, the initial administration of RTX can yield complete depletion of CD20+ B cells in the PB within 3–7 days thereafter. Multiplication to normal levels in the PB would require at least 6–12 months and 3 years in some cases (Marshall et al., 2017). Repopulation affects clinical course and outcome, and in some cases requires continued treatment, dependent upon the profiles of depletion and clearance by RTX as well as the regenerative capacity of BM. Of note, in the presence of comorbidities, especially concurrent autoimmune diseases in which several subpopulations of memory cells are formed within and without germinal centers, with different phenotypes developing (Roll et al., 2008). In autoimmune diseases, large expansions in IgD− IgM+ CD27+ and IgG− CD27+ phenotypes, which dominantly employ IgG1 and IgG3, the powerful activators of complement and are implicated in target destruction by ADCC (Berkowska et al., 2011). The process of repopulation of CD27+ IgD−IgM−CD38+ plasmablasts consists of differentiation, maturation, somatic mutation, and eventual development of plasma cells (Roll et al., 2006). However, CD20− plasma cells which are undetectable in the PB during depletion are present in the course of repopulation (Teng et al., 2012). Occasionally, pathological autoantibodies are detectable in the case of restoration to pretreatment level, since long-lived plasma cells multiply as the result of elevated generation of BAFF in the spleen. Paradoxically, RTX depletes CD20+ B cells while facilitating the differentiation and development of short-lived plasma cells to long-lived plasma cells in the spleen (Mahévas et al., 2015).

Memory B cells are vital in the clinical responses, and final clinical outcome, particularly relapse. Depletion of CD19+, CD27+ cells from the PB and BM may contribute to clinical response, which could be predictable as per the pretreatment levels of CD27+ memory cells, with better responses identified in patients with underexpression versus those with overexpression. In addition, the pre- and post-treatment levels of long-lived plasma cells and levels of survival factors are also contributory. Following RTX medication, naïve B cells expressing CD38 and CD27 repopulate, whereas the populations of non-class-switched (IgD+, CD27+) and class-switched (IgD− CD27+) memory B cells decrease (Muhammad et al., 2009). A gradual decline in levels of naïve B cells and a progressive elevation of CD27+ memory B cells is evidenced with the subsidence of the pharmacology of RTX. Accordingly, levels of plasmablasts/plasma cells may be elevated in patients with poor clinical responses to prior therapy with RTX. Other cycles could enhance the chances of beneficial clinical response prior to complete repopulation (Cambridge et al., 2014). Some patients may present with autoantibodies in clinical remission owing to long-lived plasma cells, which remain intact independent of RTX. Subsequent to depletion, B cell compartments are restored. In parallel, restoration and equilibrium in the ratios of Th1/Th2 multiply the helper T cells and T regulatory (Treg) cells.

### T Lymphocytic Responses

Recent studies have reported a significant reduction of T cells (mainly CD4+ cells) after rituximab medication in most patients, with positive correlation between CD4+ cell consumption and the clinical outcome. Accordingly, it is plausible to deem CD4+ count as a biomarker of rituximab efficacy in evaluation of clinical efficacy, since RTX is B-cell targeted, the diminished count of CD4+ cells can be attributable to the absence of antigen expression and costimulation of B cells. Facudo Fiocca Vernengo *et al.* described a mouse model of intracellular *Trypanosoma cruzi* infection and reported the efficacy of anti-CD20 therapy on responses of the B cells and CD8+ T cells, with the latter as the essential immune effectors against intracellular pathogens (Fiocca Vernengo et al., 2020).

Anti-CD20 impairs the population and function of cytotoxic T cells in a direct or indirect manner, and this defective response is regulated by the cytokine IL-17A, a cytokine that can reverse the adverse reactions of CD8+ T cells. This discovery may highlight a novel regimen for improving the compromised immunity render by depletion of B cells (Fiocca Vernengo et al., 2020). B cells can reportedly affect CD8+ T cells, with the mediators involved not elucidated whatsoever. A study also suggested that the RTX therapy is associated with the reduction of IL-17 levels in the salivary glands (SGs) in pSS patients, with the isolated mast cells potently driving Th17 polarization (Ciccia et al., 2014).

IL-22 is involved in the pSS pathogenesis, wherein the IL-22/IL-22R pathway is implicated in the development of T and B-cell lymphoma (Gelebart et al., 2011). Preliminary results of a study suggested that in the case of inflammation of salivary glands, the immunological micro-environment in pSS patients with reduced local IL-22 expression may be modified(Ciccia et al., 2013). There is a potential correlation between the RTX-dependent decrease of IL-22 expression and the lowered progression risk of pSS towards lymphoma.

In effect, the presence of Ig-secreting cell populations in the parotid salivary glands in pSS patients may avoid depletion. The surviving cells from RTX therapy may eventually explicate the disease recurrence in pSS patients undergoing RTX therapy (Hamza et al., 2012).

### CLINICAL SAFETY AND EFFICIENCY OF RITUXIMAB IN PRIMARY SJӧGREN’S SYNDROME

RTX is a chimeric monoclonal antibody directed against the pan-B lymphocyte antigen CD20, and is indicated for diseases such as leukemia, lymphoma and rheumatoid arthritis. For 2 decades, a number of high-quality clinical trials have been focused on the safety and efficacy of B-cell depletion with RTX in patients with pSS (Table 1). The patients recruited from these clinical trials were all eligible for the American-European consensus criteria for pSS. Some case reports have reported the benefit of RTX in clinical evaluation of both SS and MALT lymphoma in patients with SS (Shih et al., 2002; Somer et al., 2003).

**TABLE 1:** Main clinical trials evaluating the safety and efficacy of rituximab treatment in pSS patients.

Author (year)	Type of study	patients (special indications) (median disease duration)	Dosing frequency	Other treatments	Evaluation timepoints (follow up)	Primary outcome	Secondary outcome	Conclusion	Salivary gland function	Lacrimal gland function	RF levels	B Cells	T Cells	Other biochemical indicators	Subjective measurements	Extralandular manifestations	ESSDAI	Adverse effects
J. Pijpe et al. (2005)	prospective, single center, Open-Label, phase II	early Pss (IgG>15 g/liter, the presence of both IgM-RF and anti-SSA or anti-SSB antibodies, disease duration<4 years):8 (2 years),MALT/pSS (localized in the parotid gland):7 (7 years)	4 infusions given once weekly (low-dose)	prednisone (25 mg intravenously), clemastine (2 mg intravenously), acetaminophen (1 g) before each infusion	baseline, 5 and 12 weeks after the first infusion (12 weeks)	not well definded	not well definded	effective	stimulated submandibular/sublingual salivary secretion in patients with WSSF>0.1 ml/min:↑; Na in parotid saliva:↓	Schirmer’s test: -; rose bengal score:↓; tear break-up time in early pSS:↑	IgM-RF in patients with MALT/pSS:↓	peripheral B cells:↓	CD4+in patients with early pSS ↑	IgG, IgA, IgM, and β2-microglobulin: -; monoclinal protein:↓	MFI in early pSS:↓MFI in MALT/pSS: -	NA	NA	SSR：3 early pSS
Devauchelle-Pensec et al. (2007)	prospective, Open-Label, pilot	active pSS with VAS>50 mm, suffered from pain, sicca symptom and fratigue:16 (13.3 years)	2 infusions given once weekly (low-dose)	no concomitant treatment in therapy was administered, satble symptomatic treatment of oral and ocular discomfort was provided during the follow-up	baseline,1,2 and every 4 weeks since then until 36 weeks (36 weeks)	safety and biologic effects: B cell depletion in PB and LSG, virations of IgG, IgA, and IgM; T cell counts; RF; ANA; CRP level; ESR.	clinical VAS scores:global disease activitu, pain, sicca syndrome, fatigue. 0–100-mm VAS scores:dry mounth, dry eyes, dry vagina and dry skin. Tender and swollen joint counts; tender points; unstimulated salivary flow rate; Schirmer’s test; and Van Bijsterveld scores	effective	USW: -; salivary gland focus score: -	Schirmer’s test: -	IgA-RF:↓	CD19 + B cell:↓	T helper cells: -; cytotoxic T cells: -	IgM:↓; IgA, IgG: -ANA,anti-SSA,anti-SSB,CICs,IgG,IgA: -;NK cells: -	fatigue VAS:↓; dryness VAS:↑↑; pain VAS: ↓; global disease VAS:↓; tender point/joint count:↓SF-36: -	dry cough with bronchiolitis and interstitial pneumonia: resolved	NA	SSR：1
S Dass et al. (2008)	randomised, double-blind, placebo-controlled, pilot	pSS with fatigue VAS>50 mm and positive for anti-SSA and/or anti-SSB antoantibodies: 17 (7.25 years)	2 infusions given biweekly (high-dose)	mythylprednidolone, (100 mg IV) before each infusion	baseline, 6 months and a year	20% improvement in fatigue VAS score after 6-months therapy	measures of activity: fatigue VAS, FACIT-F, SF-36, RPOFAD, RF and serum immunoglobulins	effective	UWS: -	Schirmer’s test: -	↓	↓	NA	NA	fatigue VAS:↓general health VAS:↓PROFD: difference; SF-36 (social function, mental health domain, mental component summary):↑; physical health component of SF-36: -; pain VAS: -	NA	NA	IRR：2；SRR：1
J. M. Meijer et al. (2010)	prospective,single-center, Randomized, Double-Blind, Placebo-Controlled,prospectivve, single center (NCT00363350)	pSS with a rate of secretion of SWS of≥ 0.15 ml/min and positivity for autoantibodies(IgM-RF≥10IU/ml, anti-SSA and/or anti-SSB antoantibodies): 30 (5.25 years)	2 infusions given biweekly (high-dose)	pretreated with methylprednisolone, (100 mg IV), acetaminophen (1,000 mg orally), and clemastine (2 mg IV), and received 60 mg oral prednisone on days 1 and 2, 30 mg on days 3 and 4, and 15 mg on day 5 after each infusion	baseline,5,12,24,36 and 48 weeks (48 weeks)	significant improvement in SWS	salivary/lacrimal function and immunologic and subjective variables	effective and safe	UWS↑; SWS↑; VAS↓	Schirmer’s test: ↑; BUT test: ↑; VAS↓	IgM-RF:↓↓	obsolute count↓↓	NA	NA	MFI:↓; SF-36: ↑	the number of reported symptoms of Raynaud’s phenomenon, tendomyalgia, arthralgia:↓; 4 arthritis resolved; 1 polyneuropathy improved	NA	SSR：1；(infection:11)
E. William St.Clair et al. (2013)	prospective,open-label, single-arm, phaseⅠ(NCT0012101829)	Pss with at least one systemic manifestations: 12 (8 years)	2 infusions given biweekly (high-dose)	diphenhydramine (50 mg,orally),acetaminophen (650 mg,orally), methylprednisolone (100 mg,IV) before each infusion	basline, 4,8,14,26,30,36 and 52 weeks (52 weeks)	safety: the proportion of patients with grade3/4/5 adverse event	clinical and biologic efficacy: exocrine gland function, immune system function	safety and modest clinical benefits despite effective depletion of blood B cells	UWS--; SWS--	Schirmer’s test: -; Bijsterveld score: -	--	PB CD19 + B cell:↓↓B cell related genesd:↓	blood CD3^+^, CD4^+^, and CD8+T cells: -; CD16 + CD56 + NK cells: -; CD14^+^ and CD11b monocytes	anti-Ro/SSA, anti-La/SSB: -; anti-MR3: - IRF-4, IRF-8,IFITM-1,IFI-30,IFITM-4P: difference	tongue dryness, level of thirst, level of oral discomfort: ↓; vitality scale:↑; joint pain: -; SF-36: -	NA	NA	SAE: 2
Valerie Devauchelle-Pensec at al. (2014)	Multicenter, randomized, double-blind, placebo-controlled,parallel-group (NCT00740948)	active pSS with VAS for global disease, pain, fatigue, and dryness >50 mm:120 (8 years)	2 infusions given biweekly (high-dose)	methylprednisolone (100 mg,IV) and acetaminophen (500 mg,orally)	basline,6,16 and 24 weeks	improvement of at least 30 mm in 2 of the4 VAS scores by week 24	VAS scores at week6 and 16; disease activity, systemic manifestations, treatment activity; basal salivarly flow rate; Schiemer test and van Bijsterveld scores and Chisholm grade; CPL,ESR,RF, BAFF and so on	not alleviated at week 24, but some symptoms at earlier timepoints	salivary flow rate: -	Schirmer’s test:↑	NA	NA	NA	serum IgG, IgA, IgM, β2-microglobulin levels:↓	fatigue VAS:↓; symptom of pain: -; SF-36: -	NA	↓	respiratory disorder:1
Simon J Bowman et al. (2017); Benjamin A Fisher et al. (2018)	Multicentre, randomised, double-blind, parallel-group placebo-controlled, TRACTISS (ISRCTN 65360827)	pSS with positive anti-Ro, fatigue and oral dryness: 133 (5.3 years)	4 infusions given at week 0,2,24,26 (high-dose)	pre-infusion of methylprednisolone, acetaminophen and chlorphenamine, and post-infusion oral prednisolone	basline, 16,24,36 and 48 weeks (48 weeks)	30% reduction in either oral dryness VAS or fatigue VAS	fragitue VAS, oral dryness VAS, ESSDAI, ESSPRI, USF, MLF	neither clinically or cost-effective	UWS:↑oral dryness VAS: -	mean lachrymal flow: -	NA	NA	NA	NA	fatigue VAS: -; ESSPRI: -; SF-36: -; PROFAD-SSI: -	NA	↓	serious infusion reaction:1; infections: 2

Low-dose: 375 mg/m^2^, high-dose:1 g, VAS: visual analog scales; PB: peripheral blood, LSG: labial salivary gland, IV: intravenous; SWS: stimulated whole saliva; IRR: infusion-related action; SSR: serum sickness-like reaction; RF: rheumatoid factor; ANA: antinuclear antibody; CPR: C-reactive protein; ESR: erythrocyte sedimentation rate; NA: not available; --: no statistical significant change.

### Efficacy on Exocrine Gland Function and Sicca Symptoms

Salivary gland functionality is often assessed by unstimulated whole saliva (UWS), stimulated whole saliva (SWS), and oral dryness VAS. The reports are inconsistent, with several results showing insignificant change in salivary flow rate after RTX therapy (Devauchelle-Pensec et al., 2007; Dass et al., 2008; St Clair et al., 2013; Devauchelle-Pensec et al., 2014) while a placebo-controlled, randomized, double-blind trial indicated benefit in both whole salivary flow and oral dryness VAS with a long duration of week 24 (Meijer et al., 2010). The TRACTISS trial reported that UWS secretion remained stable in the rituximab-treated patients, whereas it worsened in the placebo group, with the latter being the only confirmatory observation in the TRACTISS trial. One plausible explication for the distinction of outcomes would be that studies with null outcomes on saliva production had recruited individuals with low saliva production at baseline. As per the UWS at baseline, Meijer et al. (2010) apparently included a less heterogeneous group of patients (Meijer et al., 2010) than the TEARS trial (Devauchelle-Pensec et al., 2014). St Clair et al. (2013) even recruited patients void of UWS at baseline, and functional improvement was paradoxically achieved (St Clair et al., 2013). Intriguingly, one trial reported decreased sodium levels in the parotid saliva, suggesting that chronic sialadenitis might be alleviated (Pijpe et al., 2005). In the author’s opinion, RTX treatment may benefit salivary gland function on the grounds that many studies have demonstrated stabilized salivary gland flow rate at least by the end of treatment in contrast to the deteriorated salivary gland flow rate in pSS patients treated with placebo.

The outcomes are in conformance with the recovery of the salivary gland parenchyma (Pijpe et al., 2009; Delli et al., 2016) and the improvement of the parotid parenchyma observed in the histopathological examinations (Cornec et al., 2016b). A study evaluating specimens of salivary gland biopsy initially identified that the reduced structural redifferentiation of glandular inflammation and lymphoepithelial duct lesions (Pijpe et al., 2009). In a concurrent study (NCT00363350) of a clinical trial, the authors reported that RTX treatment reduced local B cell infiltration and facilitated the structural recovery of salivary glands, particularly the striated ducts. The population of CD20+ B cells/mm^2^ of the parenchyma at baseline, i.e., the histopathological profiles of a parotid biopsy, may potently affect the efficacy of RTX in patients with pSS (Delli et al., 2016). In addition, the structure of salivary glands may be improved, and the number of inflammatory cells may be reduced in some patients, which may imply that the size of the salivary glands and/or the regeneration of acinar and duct components is reduced, resulting in slight enlargement of the salivary glands (Jousse-Joulin et al., 2015). However, in view of the natural history of gland function progression in pSS, nearly all researches have denied the effect of any therapeutic intervention in reversal of gland dysfunction and remedy for the dry symptoms expected at an early stage (Haldorsen et al., 2008).

The ultrasound score of salivary glands was evaluated by the total ultrasound score (TUS, range 0–11). A randomized, double-blind, multi-center TRATTISS sub-study reported statistically significant improvement of the total ultrasound score (TUS) after RTX treatment versus the placebo group (Fisher et al., 2018), which suggests that salivary gland ultrasound (SGUS) is an imaging biomarker. Thus, the diagnosis of pSS has appropriate sensitivity and good specificity.

The results of Shirmer’s test differ significantly in some studies (Meijer et al., 2010; Devauchelle-Pensec et al., 2014). However, Shirmer’s test did not suffice to detect minor changes in lacrimation. Another measurement showed that Rose-Bengal score, tear break-up time, mean tear flow, Van Bijsterveld score, and lacrimal gland VAS show a significant change in the above studies, indicating an improvement in lacrimal gland function. Meanwhile, no statistical change was reported in some studies with modest improvement in salivary gland function (Devauchelle-Pensec et al., 2007; Dass et al., 2008; St Clair et al., 2013). Some investigators also reported remission of refractory anterior scleritis in pSS after RTX treatment (Ahmadi-Simab et al., 2005).

Sicca symptoms, which include oral and ocular dryness and reflect exocrine gland function, are evaluated in clinical trials, including examination of subjective measures of patients with pSS such as dryness VAS, degree of thirst, and degree of oral discomfort. With the exception of the TRACTISS study, all studies on pSS have reported positive results in relation to sicca symptoms.

### Efficacy on Systematic Manifestations

Assessment indicators of systematic manifestation include the European League Against Rheumatism Sjögren’s Syndrome disease Activity Index (ESSDAI), visual analog scale (VAS), Short Form -36 Health Survey (SF-36), tender point/joint count, and Sjögren’s Syndrome Responder Index (SSRI). RTX therapy resulted in amelioration in subjective and objective assessment of disease severity experienced in patients with residual glandular function (Pijpe et al., 2005).

The efficacy of RTX for systemic disease activity is measured as per the ESSDAI. Of note, baseline ESSDAI scores differed widely between studies. In two large RCT studies, RTX therapy had insignificant efficacy for systemic pSS as assessed by the ESSDAI (Devauchelle-Pensec et al., 2014; Bowman et al., 2017), apart from a minor distinction in ESSDAI scores at week 36 favorable to RTX (Bowman et al., 2017). The low baseline ESSDAI score could partially account for this result. In contrast, in a prospective cohort trial enlisting 28 patients with pSS (Meiners et al., 2012), the ESSDAI score was remarkably ameliorated with RTX therapy with good validity both externally and internally. Furthermore, the idiosyncrasy of these patients also explicated the distinction in study results according to the ESSDAI score. Both indices (ESSDAI and ESSDRI) are complementary and should be combined with objective measurement of dryness and biological markers of disease severity.

Divi Cornec *et al.* defined a response to SSRI-30 as a relative benefit of ≥30% in at least two in five outcome measurements and reported significantly greater improvement in mean VAS global activity and pain scores in the SSRI-30 responder group (Cornec et al., 2015). Thus, SSRI-30 can serve as a useful indicator for therapy efficacy in future trials for pSS. The TEARS study had ended prior to its primary endpoint, which however still confirmed benefit in fatigue as of 6 weeks and dryness until 16 weeks.

Despite a normal lifespan in most patients with pSS, their health-related quality of life was compromised. The relative evaluation usually employs the generic SF-36 questionnaire, which could serve as a valuable criterion for assessment of pleomorphic diseases. The result described by Devauchelle-Pensec, V was observed that the SF-36 improved after RTX treatment, which may result from the improvement in tender point and tender joint counts (Devauchelle-Pensec et al., 2007).

Lymphoma fails to respond to rituximab alone at a low dose according to a study reported by Devauchelle-Pensec, V (Devauchelle-Pensec et al., 2007). The mechanisms underlying the lymphotoxic activity of RTX comprise complement-dependent cytotoxicity, antibody-dependent cellular cytotoxicity and induced apoptosis. Despite the lower frequency, the adverse effects are still focus of concern.

### Efficacy on Biological Changes

Laboratory assessments usually include serum biochemical assays and determination of complete blood cell counts. Three studies reported the decreased levels of RF after RTX therapy. Pijpe, J. et al. reported that B cell depletion was coupled with decreased IgM-RF levels in patients with MALT/primary SS, and other trials showed a decline in most of the participants recruited (Pijpe et al., 2005; Devauchelle-Pensec et al., 2007; Meijer et al., 2010).

Devauchelle-Pensec, V. *et al.* first reported that the anti-CD20 efficacy of B cell depletion in LSGs (Devauchelle-Pensec et al., 2007). In a study, the effect was observed in one patient with MALT lymphoma (Pijpe et al., 2005). The phenomenon of B cell depletion was reported in trials that concluded its efficacy and safety of RTX regimen. E. William St. Clair *et al.* subdivided circulating B cells into six types: transitional B cells (CD38+ CD27−), mature naïve B cells (CD38++ CD27−), mature activated memory B cells (CD38+CD27+), resting memory B cells (CD38−CD27+), plasmablasts (CD38++CD27++), and double-negative B cells (CD38−CD27−). It has been reported that median serum BAFF increased subsequent to B cell depletion and thereafter restored to baseline following reconstitution of the circulating B cell pool (St Clair et al., 2013). A companion study also suggested that memory B cell clones and plasmablast clones in PB are frequent in RTX-treated SS subjects and so are the somatic mutations. Three trials additionally demonstrated the decreased level of serum IgM after therapy (Pijpe et al., 2005; Devauchelle-Pensec et al., 2007; Devauchelle-Pensec et al., 2014) while some researchers reported insignificant changes in anti-SSA and anti-SSB (Devauchelle-Pensec et al., 2007; St Clair et al., 2013).

### Efficacy on Non-Hodgkin Lymphoma

Anti-CD20 therapy proves to be imperfect. Despite the contribution of antiCD20 mAb to increased patient survival in distinct types of B cell lymphomas, disease eradication remains impractical and impracticable. Unfortunately, the majority of patients ultimately ended up with relapses. Fortunately, some of the mechanisms underlying the drug resistance have been unmasked, thereby facilitating assessment of cancer risk after RTX treatment in the future (Yonezawa et al., 2019).

### Clinical Safety: Adverse Events

Infusion reactions, such as fever, headache, fatigue, flush, pruritus and transient headache, are the most common side effects in pSS. Of note, fatigue had significantly high prevalence in the RTX group versus the placebo groups. Fortunately, these reactions were abolished or ameliorated in all patients with dose reduction or drug discontinuation, and no specific medication or hospitalization was required. In general, the infusion reactions are mild in most of the patients. Furthermore, RTX infusion-related adverse reactions are largely attributed to the extent of B-cell lysis and generation of intracellular factors, instead of the pharmaceutic per se. Of note, patients who exhibit severe incidence rate ratios (IRRs) to RTX are generally incapable of re-challenge (Levin et al., 2017). Patients with RTX intolerance owing to IRRs are more susceptible to a poor prognosis with respect to progression-free survival and overall survival provided they are excluded from further anti-CD20 therapy.

RTX administration involving the intravenous route and the initial infusion should be performed with meticulosity and duration of hours on the grounds that over 50% of patients would exhibit infusion-related adverse effects such as pyrexia, chills, and rigors. Several infusion-related adverse reactions are reported in approximately 12% of cases, including bronchospasm and hypotension, which could be life-threatening. To minimize this toxicity, routine premedication is a prerequisite prior to each infusion. The current Summary of Product Characteristics (SPC) advises premedication with an antipyretic and an antihistamine, which includes oral paracetamol plus either intravenous chlorphenamine or an oral antihistamine, such as loratadine. Subcutaneous medication of RTX, in contrast to the intravenous injection, can reportedly improve cost-effectiveness and patient experience (Davies et al., 2014).

Serum sickness is a type III delayed hypersensitivity reaction that induces immune complexes to deposit in the tissues leading to activation of the complement cascade and inflammatory reaction. Clinical symptoms are mainly presented as fever, arthralgia and rash as well as myalgia, malaise, fatigue, conjunctival hyperemia, and purpura. Other manifestations include proteinuria, hematuria, elevated inflammatory markers, high immunoglobulin levels, and reduced complement (Bayer et al., 2019). Generally, the symptoms do not occur until the 10th day after the initial infusion of RTX, whereas adverse effects in effect develop more rapidly with medication thence. Moreover, pSS patients are more susceptible to serum sickness (-like) disease compared with patients with RA and SLE. The distinction in the latter groups of patients could be attributable to their frequent exposure to intensive immunosuppressive regimens including biological agents prior to RTX medication, whereas pSS patients are less tolerant to RTX infusion. The higher susceptibility to serum sickness per se could be innate, particularly in patients with active, early and progressive stages of pSS (Meijer et al., 2010). Interestingly, HACAs (human anti-chimeric antibodies) and serum sickness-like symptoms were reported in patients with early pSS alone rather than MALT/primary SS (Pijpe et al., 2005).

To reduce the adverse effects (infusion reactions, and serum sickness), a majority of patients should undergo pretreatment or post-treatment with other medication involving prednisone, acetaminophen, methylprednisolone, acetaminophen, clemastine, diphenhydramine, and chlorphenamine. Interestingly, Devauchelle-Pensec et al. (2007) Decrease the infusion rate instead of any concomitant treatment, indicating the good tolerance of low-dose RTX infusion, independent of corticosteroid regimen (Devauchelle-Pensec et al., 2007). Herein, the medication discussed above is proper but does not necessarily have a special efficacy to relieve the side effects, since the effect of RTX therapy on infection risk is very complex in the context of RA. The list may illustrate this complexity: 1) inconsistent RTX dosing regimens in RA; 2) RA patients receiving RTX have previously undergone varied treatments, including synthetic DMARDs, biologic DMARDs, and glucocorticoids; and 3) the elevated susceptibility at baseline to infections in patients with RA predisposes more complex adverse events of RTX. These views can hopefully serve as a reference for pSS.

In brief, an elevated susceptibility to infections and mortality associated with hypogammaglobulinemia after RTX treatment has been identified, which emphasized the necessity of follow-up of patients with hypogammaglobulinemia, with immunoglobulin levels assessed prior and subsequent to RTX regimen. Given the elevated medication of RTX, clinical practitioners are supposed to be aware of RTX-related hypogammaglobulinemia. Prior to commencement of RTX regimen, routine determination of immunoglobulin levels and baseline B-cell counts are prerequisite to preclude a potential immunodeficiency. In case of hypogammaglobulinemia, close supervision of clinical infections and laboratory results should be emphasized as well as referral to a clinical immunologist for further evaluation. Subsequent to the RTX regimen, regular laboratory evaluation should be ordered to screen out patients with persistent immune dysfunction who may benefit from IgR (Barmettler et al., 2018).

Routine practice of RTX medication in pSS includes either two doses of 1,000 mg or four doses of 375 mg/m^2^, yielding similar outcomes of B-cell depletion. It also provides contraindications in RTX dosage and depletion of B cells (Cornec et al., 2016a), so are the infusion reactions. Of note, no dramatic distinction in infection rates were observed between placebo and RTX groups (Dass et al., 2008).

Other side effects and adverse reactions are also reported in studies involving gastroenteritis (Dass et al., 2008). Some patients present with ankle arthritis with swollen joint and hives of the lower limbs. J. Pijpe et al. (2005) reported two cases of purpura following the second infusion of RTX and biopsy of a purpuric lesion showed perivascular lymphocytic infiltration in the dermis and subcutis with nuclear debris (Pijpe et al., 2005).

## Discussion

### Different Outcomes Between the Studies

Various randomized clinical studies of RTX treatment of Sjögren’s syndrome lead to different treatment results. Some small-sample pSS studies have shown that anti-CD20 therapy has a certain effect, whereas the two larger RCTs (TEARS and TRATTISS) did not meet their expectations (Devauchelle-Pensec et al., 2014; Bowman et al., 2017). This difference in efficacy may be due to distinction of patient groups in different studies and the outcome parameters used. Other reasons for the inconsistency of research results can be attributed to the following points: differences between groups of patients; different indexes evaluated after treatment, including the evaluation criteria of each index, and different evaluation tools; differences in the design of each study, including the differences between single-center and multi-center recruitment. All in all, there is a lack of consistency in evaluation criteria in clinical trials, as illustrated in clinical studies. Despite certain positive results regarding the efficacy and safety of RTX in the therapy of pSS, the trials are still limited. Therefore, a standardized, verifiable and reliable clinical disease activity index is urgently needed in future researches (Vissink et al., 2012). Despite the consumption of blood B cells in the body for the efficacy of RTX, some clinical studies have not widely adopted basic immunological examination methods in the treatment of pSS with RTX. This includes regular determination of immunoglobulin levels prior and subsequent to RTX therapy. Given this circumstance, it is difficult to ascertain whether these patients have potential immune dysfunction or secondary immune deficiency.

### Potential Mechanisms of Failure and Relapses After Rituximab Treatment

The time required for clinical remission after RTX treatment depends on many factors. A key factor is the time course for the departure of CD27+ memory B cells from the BM to the arrival at the spleen and lymphatic nodes (LNs), and development of self-reaction (Roll et al., 2008). There is evidence that by 2 years after RTX, the levels of memory cells have been reduced by over 50% versus their pretreatment measurements, which suggested the requirement of prolonged duration for remissions (Anolik et al., 2007). Paradoxically, it is well documented that longer remission occurs in the case of entry of fewer memory cells into the germinal centers of LNs and spleen and development of plasmablasts and plasma cells (Rehnberg et al., 2009).

The efficacy of RTX is relatively short-lived, and patients who receive treatment often relapse. The prevalence of recurrence is closely correlated with the follow-up duration. Generally, prolonged follow-up duration would reveal higher prevalence, even as high as 70–85% (Wang et al., 2015). A single medication routinely provides validity for 9–18 months (Edwards et al., 2007). The above results indicate that in the event of RTX discontinuation, patients would be denied long-term clinical remission and the consequent recurrence, which is comparable to the context of B-cell repopulation in PB. RTX can deplete peripheral B cells but avoid CD20 plasma cells and a lesser percentage of B cells in the tissue.

Failure of RTX treatment can be attributable to the persistence of pathogenic autoantibodies secreted by plasma cells. A large majority of patients with initial response to RTX subsequently have a relapse in the process of B-cell reconstitution, as is the case of reunion between antigens and their acquainted RTX-resistant memory B cells or reinitiation of an autoimmune response by new B cells in a microenvironment favorable to a tolerance breakdown in the process of B-cell lymphopoiesis. Recurrence usually occurs after the regenerated CD20+ B cells restores to the pretreatment level. Hence, larger trials are invited to authenticate approaches to responder selection and the optimal therapeutic options so as to improve the efficacy of RTX in the regimen for pSS.

The increased entry of B cells from BM into the microenvironment (Becerra et al., 2015) would lead to the differentiation of self-reactive B cells via appropriate signaling (Cambridge et al., 2014). At the active stage of the disease, the levels of BAFF and autoreactive plasmablasts in PB increase. However, regardless of the BAFF levels in the serum, the BAFF-R expression of naïve and memory B cells at relapse would decrease. The regeneration of self-reactive memory B cells and/or plasmablasts is accompanied by relapse, which can help to predict recurrence. Soluble-free light chains and CD23 also played an essential part in the differentiation of plasmablasts which had developed at an early stage of relapse (Ehrenstein and Wing, 2016). In the course of relapse, CD95+ CD27+ cells that secrete pro-inflammatory TNFα and IL-10 have a larger proportion than transitional cells in PB (Brezinschek et al., 2012). In the microenvironment of the germinal center, persistence of memory B cells is free from T cell interaction but pivotal to their differentiation into long-lived plasma cells (Johansson-Lindbom and Borrebaeck, 2002), wherein proinflammatory autoreactive B cells express Ki67 marker on pre-B and immature B cells from the BM, whereas kappa-deleting recombination excision circles are noted for migration of transitional cells (Shahaf et al., 2016).

In addition, one of the adverse reactions in B cell depletion therapy is significant infection, which leads to sepsis and death due to immunosuppression. Fortunately, IVIg brings hope to reduce such risks. Late-onset neutropenia related to pneumonia and heart problems is worth noting. From a panoramic point of view, some researchers have proposed that anti-CD20 therapies currently available cannot restore the patient to a prior immunity akin to the ‘tabula rasa’ (i.e., a blank slate), wherein all the prior levels reminiscent of memory and proof of (auto) immune responses have been eradicated. Processes that are critical to central and peripheral tolerance, such as energy deficiency, receptor edition and deletion, are free from RTX, thereby unable to annul autoimmunity. Consequently, autoreactive B cells produce inflammation hand in hand with autoreactive T cells, leading to pathological changes in the microenvironment and recurrence of autoimmune diseases.

### Limitations of Rituximab Monotherapy

Despite the safety of RTX to date, evidence of the long-term efficacy and safety of RTX “monotherapy” in pSS is still scarce. Before approval of the RTX medication combined with other biological products, a large-scale randomized controlled trial of pSS patients with prolonged follow-up is prerequisite. In theory, the combination therapy of RTX and other biological agents should be beneficial, such as a combined therapy targeting CD20 (RTX) and BAFF. In addition, to our knowledge, for certain pSS patients with severe extraglandular manifestations (such as vasculitis, nephritis, or polyneuropathy), RTX alone is far from enough. Therefore, the regimens for these individuals would require more potent immunosuppressive therapy.

Another drawback of RTX therapy is not the mere adverse autoimmune response affected, but the beneficial humoral response as well. Despite the vague pathogenic mechanism of autoantibodies and incomplete knowledge of all autoantigens, the onset of tolerance to autoreactive B cells will be delightful. Hence, autoantigen-specific B cell-targeted therapies would bring prospect, by depleting or silencing pathogenic self-antigen-reactive cells whilst retaining B cells required for immune defense. More frequently, it is the differentiated daughters of autoreactive B cells that generate autoantibodies and mediate the disease. Nevertheless, regimens with pan-B cell depleting agents, such as RTX and belimumab (anti-BAFF), are imperfect on the grounds that these agents are non-specifically targeted, and cannot discriminate pathogenic B cells from non-pathogenic, thus leading to the comprehensive suppression of humoral immunity. Although prior immunity could be retained, the newly developed immunity would be invalidated in the encounter with pathogenic strangers, such as SARS-CoV-2. Moreover, despite the typical duration of 6–12 months of B cell depletion following treatment termination, patients in some cases had never stored to their initial B cell population (Venhoff et al., 2014). Finally, anti-CD20 regimens target most of B cell subsets merely retain antibody-producing plasma cells, without expressing CD20 on their cell surface. This finding may explicate, at least in part, the inconsistent responses to therapy for diseases such as SLE and rheumatoid arthritis (RA), wherein autoantibodies might be incompletely reduced. Therefore, the optimal antigen-specific therapies would be those targeting the pathogenic B cells including plasma cells, and meanwhile rendering the immune system function intact.

### Combination Therapy to Improve Rituximab Efficacy

To date, one of the challenges in pSS treatment is the lack of optimal strategies to prevent lymphoma in patients at high risk of SS and to effectively manage SS-related lymphoma. The efficacy of anti-CD20 therapy on marginal zone B cells in the murine model for human CD20 expression was identified only before anti-BAFF treatment (Gong et al., 2005). Belimumab, a monoclonal anti-BAFF antibody against human BLyS protein, is a registered agent in the therapy for SLE. It is worth noting that two clinical studies are currently underway to verify the effects of rituximab-belimumab as a sequential therapy for SLE (NCT02260934 and NCT02284984 on clinicaltrials.org). As for further strategies for pSS therapy, a report described that belimumab combined with RTX may be useful for SS-related B-cell lymphoproliferation and overexpressed B-cell activating factor (BAFF) in MALT. It may reduce the effect of RTX in the therapy of SS. Furthermore, no related side effects were reported after continuous negative serum cryoglobulin and rheumatoid factor treatment, suggesting efficacy and safety (De Vita et al., 2014). In this case, a multi-center, double-blind, randomized clinical trial (RCT) is underway (NCT02631538 on clinicaltrials.org). In one pSS patient with refractory cryoglobulinemia vasculitis comorbid with low-grade MALT lymphoma, the combined regimen of belimumab and rituximab yielded dramatic improvement of vasculitis and complete remission of lymphoma (Chen et al., 2016).

According to Advani and others, CD47-blocking antibody Hu5F9-G4 in combination with RTX can benefit patients with non-Hodgkin’s lymphoma (Advani et al., 2018). The anti-tumor efficacy of anti-CD20 therapy is reportedly related to antibody-dependent cytotoxicity (ADCC), including NK cells and macrophages and the involvement of CD8+ and CD4+ T cells. Hence, the combination of RTX and IL-2 has undergone preclinical testing and clinical trials (Eisenbeis et al., 2004; Casadesús et al., 2020). Other researchers have developed an antibody-drug conjugate (ADC) rituximab-vcMMAE, which delivers highly cytotoxic drugs directly to CD20-positive cells to abate RTX resistance and elevate the efficacy of RTX (Abdollahpour-Alitappeh et al., 2018). In addition, with respect to patients who are intolerant to RTX, ofatumumab is a completely humanized anti-CD20 monoclonal antibody, which has been confirmed to be safe and may be a valuable alternative for patients with RTX intolerance (Chen et al., 2019). However, further research is still required to assess the effectiveness and safety of ofatumumab in combination with RTX.

## Conclusion

The option of therapeutic agents for autoimmune diseases should be centered around the need for explicit solutions to scientific fact-based, predictive indicators of progression, prognosis, promise of long-lasting clinical remission free from further treatment as well as safety, paucity of immediate and long-term adverse events, facility in access, simplicity in management and guaranty of a good quality of life. With the advent of novel targeted therapies, knowledge of evaluation of patients with pSS will increase, which will promote the conception of clinical trials and the establishment of effective therapeutic options for pSS. Thus, RTX is valuable for specific subgroups of pSS patients and serves as a general regimen for pSS.

## References

[B1] Abdollahpour-Alitappeh M. Hashemi Karouei S. M. Lotfinia M. Amanzadeh A. Habibi-Anbouhi M. (2018). A Developed Antibody-Drug Conjugate Rituximab-vcMMAE Shows a Potent Cytotoxic Activity against CD20-Positive Cell Line. Artif. Cell Nanomed Biotechnol 46 (Suppl. 2), 1–8. 10.1080/21691401.2018.1449119 29523024

[B2] Advani R. Flinn I. Popplewell L. Forero A. Bartlett N. L. Ghosh N. (2018). CD47 Blockade by Hu5F9-G4 and Rituximab in Non-hodgkin's Lymphoma. N. Engl. J. Med. 379 (18), 1711–1721. 10.1056/NEJMoa1807315 30380386 PMC8058634

[B3] Ahmadi-Simab K. Lamprecht P. Nölle B. Ai M. Gross W. L. (2005). Successful Treatment of Refractory Anterior Scleritis in Primary Sjogren's Syndrome with Rituximab. Ann. Rheum. Dis. 64 (7), 1087–1088. 10.1136/ard.2004.027128 15958765 PMC1755562

[B4] Anolik J. H. Barnard J. Owen T. Zheng B. Kemshetti S. Looney R. J. (2007). Delayed Memory B Cell Recovery in Peripheral Blood and Lymphoid Tissue in Systemic Lupus Erythematosus after B Cell Depletion Therapy. Arthritis Rheum. 56 (9), 3044–3056. 10.1002/art.22810 17763423

[B5] Barmettler S. Ong M. S. Farmer J. R. Choi H. Walter J. (2018). Association of Immunoglobulin Levels, Infectious Risk, and Mortality with Rituximab and Hypogammaglobulinemia. JAMA Netw. Open 1 (7), e184169. 10.1001/jamanetworkopen.2018.4169 30646343 PMC6324375

[B6] Barr T. A. Shen P. Brown S. Lampropoulou V. Roch T. Lawrie S. (2012). B Cell Depletion Therapy Ameliorates Autoimmune Disease through Ablation of IL-6-producing B Cells. J. Exp. Med. 209 (5), 1001–1010. 10.1084/jem.20111675 22547654 PMC3348102

[B7] Bayer G. Agier M. S. Lioger B. Lepelley M. Zenut M. Lanoue M. C. (2019). Rituximab-induced Serum Sickness Is More Frequent in Autoimmune Diseases as Compared to Hematological Malignancies: A French Nationwide Study. Eur. J. Intern. Med. 67, 59–64. 10.1016/j.ejim.2019.06.009 31279430

[B8] Becerra E. Scully M. A. Leandro M. J. Heelas E. O. Westwood J. P. De La Torre I. (2015). Effect of Rituximab on B Cell Phenotype and Serum B Cell-Activating Factor Levels in Patients with Thrombotic Thrombocytopenic Purpura. Clin. Exp. Immunol. 179 (3), 414–425. 10.1111/cei.12472 25339550 PMC4337674

[B9] Beers S. A. Chan C. H. French R. R. Cragg M. S. Glennie M. J. (2010). CD20 as a Target for Therapeutic Type I and II Monoclonal Antibodies. Semin. Hematol. 47 (2), 107–114. 10.1053/j.seminhematol.2010.01.001 20350657

[B10] Berkowska M. A. Driessen G. J. Bikos V. Grosserichter-Wagener C. Stamatopoulos K. Cerutti A. (2011). Human Memory B Cells Originate from Three Distinct Germinal center-dependent and -independent Maturation Pathways. Blood 118 (8), 2150–2158. 10.1182/blood-2011-04-345579 21690558 PMC3342861

[B11] Bowman S. J. Everett C. C. O'Dwyer J. L. Emery P. Pitzalis C. Ng W. F. (2017). Randomized Controlled Trial of Rituximab and Cost-Effectiveness Analysis in Treating Fatigue and Oral Dryness in Primary Sjögren's Syndrome. Arthritis Rheumatol. 69 (7), 1440–1450. 10.1002/art.40093 28296257

[B12] Brezinschek H. P. Rainer F. Brickmann K. Graninger W. B. (2012). B Lymphocyte-Typing for Prediction of Clinical Response to Rituximab. Arthritis Res. Ther. 14 (4), R161. 10.1186/ar3901 22770118 PMC3580553

[B13] Cambridge G. Perry H. C. Nogueira L. Serre G. Parsons H. M. De La Torre I. (2014). The Effect of B-Cell Depletion Therapy on Serological Evidence of B-Cell and Plasmablast Activation in Patients with Rheumatoid Arthritis over Multiple Cycles of Rituximab Treatment. J. Autoimmun. 50, 67–76. 10.1016/j.jaut.2013.12.002 24365380

[B14] Casadesús A. V. Deligne C. Diallo B. K. Sosa K. Josseaume N. Mesa C. (2020). A Rationally-Engineered IL-2 Improves the Antitumor Effect of Anti-CD20 Therapy. Oncoimmunology 9 (1), 1770565. 10.1080/2162402x.2020.1770565 32923126 PMC7458652

[B15] Chen D. Taylor K. P. Hall Q. Kaplan J. M. (2016). The Neuropeptides FLP-2 and PDF-1 Act in Concert to Arouse *Caenorhabditis elegans* Locomotion. Genetics 204 (3), 1151–1159. 10.1534/genetics.116.192898 27585848 PMC5105848

[B16] Chen L. Y. Shah R. Cwynarski K. Lambert J. McNamara C. Mohamedbhai S. G. (2019). Ofatumumab Is a Feasible Alternative Anti-CD20 Therapy in Patients Intolerant of Rituximab. Br. J. Haematol. 184 (3), 462–465. 10.1111/bjh.15110 29363752

[B17] Ciccia F. Giardina A. Rizzo A. Guggino G. Cipriani P. Carubbi F. (2013). Rituximab Modulates the Expression of IL-22 in the Salivary Glands of Patients with Primary Sjogren's Syndrome. Ann. Rheum. Dis. 72 (5), 782–783. 10.1136/annrheumdis-2012-202754 23264342

[B18] Ciccia F. Guggino G. Rizzo A. Alessandro R. Carubbi F. Giardina A. (2014). Rituximab Modulates IL-17 Expression in the Salivary Glands of Patients with Primary Sjögren's Syndrome. Rheumatology (Oxford) 53 (7), 1313–1320. 10.1093/rheumatology/keu004 24602921

[B19] Cornec D. Costa S. Devauchelle-Pensec V. Jousse-Joulin S. Marcorelles P. Berthelot J. M. (2016a). Blood and Salivary-Gland BAFF-Driven B-Cell Hyperactivity Is Associated to Rituximab Inefficacy in Primary Sjögren's Syndrome. J. Autoimmun. 67, 102–110. 10.1016/j.jaut.2015.11.002 26688003

[B20] Cornec D. Devauchelle-Pensec V. Mariette X. Jousse-Joulin S. Berthelot J. M. Perdriger A. (2015). Development of the Sjögren's Syndrome Responder Index, a Data-Driven Composite Endpoint for Assessing Treatment Efficacy. Rheumatology (Oxford) 54 (9), 1699–1708. 10.1093/rheumatology/kev114 25957440

[B21] Cornec D. Jousse-Joulin S. Costa S. Marhadour T. Marcorelles P. Berthelot J. M. (2016b). High-Grade Salivary-Gland Involvement, Assessed by Histology or Ultrasonography, Is Associated with a Poor Response to a Single Rituximab Course in Primary Sjögren's Syndrome: Data from the TEARS Randomized Trial. PLoS One 11 (9), e0162787. 10.1371/journal.pone.0162787 27662653 PMC5035078

[B22] Dass S. Bowman S. J. Vital E. M. Ikeda K. Pease C. T. Hamburger J. (2008). Reduction of Fatigue in Sjögren Syndrome with Rituximab: Results of a Randomised, Double-Blind, Placebo-Controlled Pilot Study. Ann. Rheum. Dis. 67 (11), 1541–1544. 10.1136/ard.2007.083865 18276741

[B23] Davies A. Merli F. Mihaljevic B. Siritanaratkul N. Solal-Céligny P. Barrett M. (2014). Pharmacokinetics and Safety of Subcutaneous Rituximab in Follicular Lymphoma (SABRINA): Stage 1 Analysis of a Randomised Phase 3 Study. Lancet Oncol. 15 (3), 343–352. 10.1016/s1470-2045(14)70005-1 24521993

[B24] De Vita S. Quartuccio L. Salvin S. Picco L. Scott C. A. Rupolo M. (2014). Sequential Therapy with Belimumab Followed by Rituximab in Sjögren's Syndrome Associated with B-Cell Lymphoproliferation and Overexpression of BAFF: Evidence for Long-Term Efficacy. Clin. Exp. Rheumatol. 32 (4), 490–494. 24802131

[B25] Deans J. P. Robbins S. M. Polyak M. J. Savage J. A. (1998). Rapid Redistribution of CD20 to a Low Density Detergent-Insoluble Membrane Compartment. J. Biol. Chem. 273 (1), 344–348. 10.1074/jbc.273.1.344 9417086

[B26] Delli K. Haacke E. A. Kroese F. G. Pollard R. P. Ihrler S. van der Vegt B. (2016). Towards Personalised Treatment in Primary Sjögren's Syndrome: Baseline Parotid Histopathology Predicts Responsiveness to Rituximab Treatment. Ann. Rheum. Dis. 75 (11), 1933–1938. 10.1136/annrheumdis-2015-208304 26757748

[B27] Devauchelle-Pensec V. Mariette X. Jousse-Joulin S. Berthelot J. M. Perdriger A. Puéchal X. (2014). Treatment of Primary Sjögren Syndrome with Rituximab: a Randomized Trial. Ann. Intern. Med. 160 (4), 233–242. 10.7326/m13-1085 24727841

[B28] Devauchelle-Pensec V. Pennec Y. Morvan J. Pers J. O. Daridon C. Jousse-Joulin S. (2007). Improvement of Sjögren's Syndrome after Two Infusions of Rituximab (Anti-CD20). Arthritis Rheum. 57 (2), 310–317. 10.1002/art.22536 17330280

[B29] Edwards J. C. Cambridge G. Leandro M. J. (2007). Repeated B-Cell Depletion in Clinical Practice. Rheumatology (Oxford) 46 (9), 1509. 10.1093/rheumatology/kem164 17623746

[B30] Ehrenstein M. R. Wing C. (2016). The BAFFling Effects of Rituximab in Lupus: Danger Ahead? Nat. Rev. Rheumatol. 12 (6), 367–372. 10.1038/nrrheum.2016.18 26888554

[B31] Einfeld D. A. Brown J. P. Valentine M. A. Clark E. A. Ledbetter J. A. (1988). Molecular Cloning of the Human B Cell CD20 Receptor Predicts a Hydrophobic Protein with Multiple Transmembrane Domains. Embo j 7 (3), 711–717. 10.1002/j.1460-2075.1988.tb02867.x 2456210 PMC454379

[B32] Eisenbeis C. F. Grainger A. Fischer B. Baiocchi R. A. Carrodeguas L. Roychowdhury S. (2004). Combination Immunotherapy of B-Cell Non-hodgkin's Lymphoma with Rituximab and Interleukin-2: a Preclinical and Phase I Study. Clin. Cancer Res. 10 (18 Pt 1), 6101–6110. 10.1158/1078-0432.Ccr-04-0525 15447996

[B33] Fiocca Vernengo F. Beccaria C. G. Araujo Furlan C. L. Tosello Boari J. Almada L. Gorosito Serrán M. (2020). CD8+ T Cell Immunity Is Compromised by Anti-CD20 Treatment and Rescued by Interleukin-17A. mBio 11 (3). 10.1128/mBio.00447-20 PMC7218282 32398312

[B34] Fisher B. A. Everett C. C. Rout J. O'Dwyer J. L. Emery P. Pitzalis C. (2018). Effect of Rituximab on a Salivary Gland Ultrasound Score in Primary Sjögren's Syndrome: Results of the TRACTISS Randomised Double-Blind Multicentre Substudy. Ann. Rheum. Dis. 77 (3), 412–416. 10.1136/annrheumdis-2017-212268 29275334 PMC5867400

[B35] Galarza C. Valencia D. Tobón G. J. Zurita L. Mantilla R. D. Pineda-Tamayo R. (2008). Should Rituximab Be Considered as the First-Choice Treatment for Severe Autoimmune Rheumatic Diseases? Clin. Rev. Allergy Immunol. 34 (1), 124–128. 10.1007/s12016-007-8028-z 18270866

[B36] Gelebart P. Zak Z. Dien-Bard J. Anand M. Lai R. (2011). Interleukin 22 Signaling Promotes Cell Growth in Mantle Cell Lymphoma. Transl Oncol. 4 (1), 9–19. 10.1593/tlo.10172 21286373 PMC3026902

[B37] Gong Q. Ou Q. Ye S. Lee W. P. Cornelius J. Diehl L. (2005). Importance of Cellular Microenvironment and Circulatory Dynamics in B Cell Immunotherapy. J. Immunol. 174 (2), 817–826. 10.4049/jimmunol.174.2.817 15634903

[B38] Gottenberg J. E. Seror R. Miceli-Richard C. Benessiano J. Devauchelle-Pensec V. Dieude P. (2013). Serum Levels of Beta2-Microglobulin and Free Light Chains of Immunoglobulins Are Associated with Systemic Disease Activity in Primary Sjögren's Syndrome. Data at Enrollment in the Prospective ASSESS Cohort. PLoS One 8 (5), e59868. 10.1371/journal.pone.0059868 23717383 PMC3663789

[B39] Groom J. Kalled S. L. Cutler A. H. Olson C. Woodcock S. A. Schneider P. (2002). Association of BAFF/BLyS Overexpression and Altered B Cell Differentiation with Sjögren's Syndrome. J. Clin. Invest. 109 (1), 59–68. 10.1172/jci14121 11781351 PMC150825

[B40] Gürcan H. M. Keskin D. B. Stern J. N. Nitzberg M. A. Shekhani H. Ahmed A. R. (2009). A Review of the Current Use of Rituximab in Autoimmune Diseases. Int. Immunopharmacol 9 (1), 10–25. 10.1016/j.intimp.2008.10.004 19000786

[B41] Haldorsen K. Moen K. Jacobsen H. Jonsson R. Brun J. G. (2008). Exocrine Function in Primary Sjögren Syndrome: Natural Course and Prognostic Factors. Ann. Rheum. Dis. 67 (7), 949–954. 10.1136/ard.2007.074203 17962240

[B42] Hamza N. Bootsma H. Yuvaraj S. Spijkervet F. K. Haacke E. A. Pollard R. P. (2012). Persistence of Immunoglobulin-Producing Cells in Parotid Salivary Glands of Patients with Primary Sjögren's Syndrome after B Cell Depletion Therapy. Ann. Rheum. Dis. 71 (11), 1881–1887. 10.1136/annrheumdis-2011-201189 22615459

[B43] Johansson-Lindbom B. Borrebaeck C. A. (2002). Germinal center B Cells Constitute a Predominant Physiological Source of IL-4: Implication for Th2 Development *In Vivo* . J. Immunol. 168 (7), 3165–3172. 10.4049/jimmunol.168.7.3165 11907068

[B44] Jousse-Joulin S. Devauchelle-Pensec V. Cornec D. Marhadour T. Bressollette L. Gestin S. (2015). Brief Report: Ultrasonographic Assessment of Salivary Gland Response to Rituximab in Primary Sjögren's Syndrome. Arthritis Rheumatol. 67 (6), 1623–1628. 10.1002/art.39088 25708147

[B45] Lavie F. Miceli-Richard C. Ittah M. Sellam J. Gottenberg J. E. Mariette X. (2007). Increase of B Cell-Activating Factor of the TNF Family (BAFF) after Rituximab Treatment: Insights into a New Regulating System of BAFF Production. Ann. Rheum. Dis. 66 (5), 700–703. 10.1136/ard.2006.060772 17040963 PMC1954605

[B46] Levin A. S. Otani I. M. Lax T. Hochberg E. Banerji A. (2017). Reactions to Rituximab in an Outpatient Infusion Center: A 5-Year Review. J. Allergy Clin. Immunol. Pract. 5 (1), 107–e1. 10.1016/j.jaip.2016.06.022 27497683

[B48] Mahévas M. Michel M. Vingert B. Moroch J. Boutboul D. Audia S. (2015). Emergence of Long-Lived Autoreactive Plasma Cells in the Spleen of Primary Warm Auto-Immune Hemolytic Anemia Patients Treated with Rituximab. J. Autoimmun. 62, 22–30. 10.1016/j.jaut.2015.05.006 26112660

[B49] Maloney D. G. Smith B. Rose A. (2002). Rituximab: Mechanism of Action and Resistance. Semin. Oncol. 29 (1s2), 2–9. 10.1053/sonc.2002.30156 28140087

[B50] Mariette X. Criswell L. A. (2018). Primary Sjögren's Syndrome. N. Engl. J. Med. 379 (10), 97–939. 10.1056/NEJMcp170251410.1056/NEJMc1804598 29972746

[B51] Marshall M. J. E. Stopforth R. J. Cragg M. S. (2017). Therapeutic Antibodies: What Have We Learnt from Targeting CD20 and where Are We Going? Front. Immunol. 8, 1245. 10.3389/fimmu.2017.01245 29046676 PMC5632755

[B52] Mavragani C. P. Fragoulis G. E. Rontogianni D. Kanariou M. Moutsopoulos H. M. (2014). Elevated IgG4 Serum Levels Among Primary Sjögren's Syndrome Patients: Do They Unmask Underlying IgG4-Related Disease? Arthritis Care Res. (Hoboken) 66 (15), 773–777. 10.1503/cmaj.12203710.1002/acr.22216 25100215

[B53] Mei H. E. Wirries I. Frölich D. Brisslert M. Giesecke C. Grün J. R. (2015). A Unique Population of IgG-Expressing Plasma Cells Lacking CD19 Is Enriched in Human Bone Marrow. Blood 125 (11), 1739–1748. 10.1182/blood-2014-02-555169 25573986

[B54] Meijer J. M. Meiners P. M. Vissink A. Spijkervet F. K. Abdulahad W. Kamminga N. (2010). Effectiveness of Rituximab Treatment in Primary Sjögren's Syndrome: a Randomized, Double-Blind, Placebo-Controlled Trial. Arthritis Rheum. 62 (4), 960–968. 10.1002/art.27314 20131246

[B55] Meiners P. M. Arends S. Brouwer E. Spijkervet F. K. Vissink A. Bootsma H. (2012). Responsiveness of Disease Activity Indices ESSPRI and ESSDAI in Patients with Primary Sjögren's Syndrome Treated with Rituximab. Ann. Rheum. Dis. 71 (8), 1297–1302. 10.1136/annrheumdis-2011-200460 22258489

[B56] Muhammad K. Roll P. Einsele H. Dörner T. Tony H. P. (2009). Delayed Acquisition of Somatic Hypermutations in Repopulated IGD+CD27+ Memory B Cell Receptors after Rituximab Treatment. Arthritis Rheum. 60 (8), 2284–2293. 10.1002/art.24722 19644860

[B57] Nagel A. Podstawa E. Eickmann M. Müller H. H. Hertl M. Eming R. (2009). Rituximab Mediates a strong Elevation of B-Cell-Activating Factor Associated with Increased Pathogen-specific IgG but Not Autoantibodies in Pemphigus Vulgaris. J. Invest. Dermatol. 129 (9), 2202–2210. 10.1038/jid.2009.27 19282839

[B59] Pérez-Callejo D. González-Rincón J. Sánchez A. Provencio M. Sánchez-Beato M. (2015). Action and Resistance of Monoclonal CD20 Antibodies Therapy in B-Cell Non-hodgkin Lymphomas. Cancer Treat. Rev. 41 (8), 680–689. 10.1016/j.ctrv.2015.05.007 26045227

[B60] Pijpe J. Meijer J. M. Bootsma H. van der Wal J. E. Spijkervet F. K. Kallenberg C. G. (2009). Clinical and Histologic Evidence of Salivary Gland Restoration Supports the Efficacy of Rituximab Treatment in Sjögren's Syndrome. Arthritis Rheum. 60 (11), 3251–3256. 10.1002/art.24903 19877054

[B61] Pijpe J. van Imhoff G. W. Spijkervet F. K. Roodenburg J. L. Wolbink G. J. Mansour K. (2005). Rituximab Treatment in Patients with Primary Sjögren's Syndrome: an Open-Label Phase II Study. Arthritis Rheum. 52 (9), 2740–2750. 10.1002/art.21260 16142737

[B62] Pollard R. P. Abdulahad W. H. Vissink A. Hamza N. Burgerhof J. G. Meijer J. M. (2013). Serum Levels of BAFF, but Not APRIL, Are Increased after Rituximab Treatment in Patients with Primary Sjogren's Syndrome: Data from a Placebo-Controlled Clinical Trial. Ann. Rheum. Dis. 72 (1), 146–148. 10.1136/annrheumdis-2012-202071 22851468

[B63] Ramos-Casals M. Brito-Zerón P. Bombardieri S. Bootsma H. De Vita S. Dörner T. (2020). EULAR Recommendations for the Management of Sjögren's Syndrome with Topical and Systemic Therapies. Ann. Rheum. Dis. 79 (1), 3–18. 10.1136/annrheumdis-2019-216114 31672775

[B64] Ramos-Casals M. Brito-Zerón P. Sisó-Almirall A. Bosch X. Tzioufas A. G. (2012). Topical and Systemic Medications for the Treatment of Primary Sjögren's Syndrome. Nat. Rev. Rheumatol. 8 (7), 399–411. 10.1038/nrrheum.2012.53 22549247

[B65] Rehnberg M. Amu S. Tarkowski A. Bokarewa M. I. Brisslert M. (2009). Short- and Long-Term Effects of Anti-CD20 Treatment on B Cell Ontogeny in Bone Marrow of Patients with Rheumatoid Arthritis. Arthritis Res. Ther. 11 (4), R123. 10.1186/ar2789 19686595 PMC2745807

[B66] Roll P. Dörner T. Tony H. P. (2008). Anti-CD20 Therapy in Patients with Rheumatoid Arthritis: Predictors of Response and B Cell Subset Regeneration after Repeated Treatment. Arthritis Rheum. 58 (6), 1566–1575. 10.1002/art.23473 18512772

[B67] Roll P. Palanichamy A. Kneitz C. Dorner T. Tony H. P. (2006). Regeneration of B Cell Subsets after Transient B Cell Depletion Using Anti-CD20 Antibodies in Rheumatoid Arthritis. Arthritis Rheum. 54 (8), 2377–2386. 10.1002/art.22019 16869000

[B68] Schneider P. (2005). The Role of APRIL and BAFF in Lymphocyte Activation. Curr. Opin. Immunol. 17 (3), 282–289. 10.1016/j.coi.2005.04.005 15886118

[B69] Seror R. Ravaud P. Bowman S. J. Baron G. Tzioufas A. Theander E. (2010). EULAR Sjogren's Syndrome Disease Activity index: Development of a Consensus Systemic Disease Activity index for Primary Sjogren's Syndrome. Ann. Rheum. Dis. 69 (6), 1103–1109. 10.1136/ard.2009.110619 19561361 PMC2937022

[B70] Seror R. Ravaud P. Mariette X. Bootsma H. Theander E. Hansen A. (2011). EULAR Sjogren's Syndrome Patient Reported Index (ESSPRI): Development of a Consensus Patient index for Primary Sjogren's Syndrome. Ann. Rheum. Dis. 70 (6), 968–972. 10.1136/ard.2010.143743 21345815

[B71] Shahaf G. Zisman-Rozen S. Benhamou D. Melamed D. Mehr R. (2016). B Cell Development in the Bone Marrow Is Regulated by Homeostatic Feedback Exerted by Mature B Cells. Front. Immunol. 7, 77. 10.3389/fimmu.2016.00077 27047488 PMC4801882

[B72] Shih W. J. Ghesani N. Hongming Z. Alavi A. Schusper S. Mozley D. (2002). F-18 FDG Positron Emission Tomography Demonstrates Resolution of Non-hodgkin's Lymphoma of the Parotid Gland in a Patient with Sjogren's Syndrome: before and after Anti-CD20 Antibody Rituximab Therapy. Clin. Nucl. Med. 27 (2), 142–143. 10.1097/00003072-200202000-00019 11786752

[B73] Silverman G. J. Boyle D. L. (2008). Understanding the Mechanistic Basis in Rheumatoid Arthritis for Clinical Response to Anti-CD20 Therapy: the B-Cell Roadblock Hypothesis. Immunol. Rev. 223, 175–185. 10.1111/j.1600-065X.2008.00627.x 18613836

[B74] Somer B. G. Tsai D. E. Downs L. Weinstein B. Schuster S. J. (2003). Improvement in Sjögren's Syndrome Following Therapy with Rituximab for Marginal Zone Lymphoma. Arthritis Rheum. 49 (3), 394–398. 10.1002/art.11109 12794796

[B75] Song L. Wang Y. Zhang J. Song N. Xu X. Lu Y. (2018). The Risks of Cancer Development in Systemic Lupus Erythematosus (SLE) Patients: a Systematic Review and Meta-Analysis. Arthritis Res. Ther. 20 (1), 270. 10.1186/s13075-018-1760-3 30522515 PMC6282326

[B76] St Clair E. W. Levesque M. C. Prak E. T. Vivino F. B. Alappatt C. J. Spychala M. E. (2013). Rituximab Therapy for Primary Sjögren's Syndrome: an Open-Label Clinical Trial and Mechanistic Analysis. Arthritis Rheum. 65 (4), 1097–1106. 10.1002/art.37850 23334994 PMC3618621

[B78] Teng Y. K. Wheater G. Hogan V. E. Stocks P. Levarht E. W. Huizinga T. W. (2012). Induction of Long-Term B-Cell Depletion in Refractory Rheumatoid Arthritis Patients Preferentially Affects Autoreactive More Than Protective Humoral Immunity. Arthritis Res. Ther. 14 (2), R57. 10.1186/ar3770 22409963 PMC3446423

[B79] Vallerskog T. Heimbürger M. Gunnarsson I. Zhou W. Wahren-Herlenius M. Trollmo C. (2006). Differential Effects on BAFF and APRIL Levels in Rituximab-Treated Patients with Systemic Lupus Erythematosus and Rheumatoid Arthritis. Arthritis Res. Ther. 8 (6), R167. 10.1186/ar2076 17092341 PMC1794511

[B80] Venhoff N. Niessen L. Kreuzaler M. Rolink A. G. Hässler F. Rizzi M. (2014). Reconstitution of the Peripheral B Lymphocyte Compartment in Patients with ANCA-Associated Vasculitides Treated with Rituximab for Relapsing or Refractory Disease. Autoimmunity 47 (6), 401–408. 10.3109/08916934.2014.914174 24798501

[B81] Vissink A. Bootsma H. Spijkervet F. K. Hu S. Wong D. T. Kallenberg C. G. (2012). Current and Future Challenges in Primary Sjögren's Syndrome. Curr. Pharm. Biotechnol. 13 (10), 2026–2045. 10.2174/138920112802273254 22208656

[B82] Wang H. H. Liu C. W. Li Y. C. Huang Y. C. (2015). Efficacy of Rituximab for Pemphigus: a Systematic Review and Meta-Analysis of Different Regimens. Acta Derm Venereol. 95 (8), 928–932. 10.2340/00015555-2116 25881672

[B83] Yonezawa A. Otani Y. Kitano T. Mori M. Masui S. Isomoto Y. (2019). Concentration and Glycoform of Rituximab in Plasma of Patients with B Cell Non-hodgkin's Lymphoma. Pharm. Res. 36 (6), 82. 10.1007/s11095-019-2624-5 30989405

